# Major depressive disorder elevates the risk of dentofacial deformity: a bidirectional two-sample Mendelian randomization study

**DOI:** 10.3389/fpsyt.2024.1442679

**Published:** 2024-07-30

**Authors:** Jinhan Nie, Yi Zhang, Jun Ma, Qing Xue, Min Hu, Huichuan Qi

**Affiliations:** ^1^ Department of Orthodontics, Hospital of Stomatology, Jilin University, Changchun, Jilin, China; ^2^ Department of Oral Anatomy and Physiology, Hospital of Stomatology, Jilin University, Changchun, Jilin, China; ^3^ Key Laboratory of Pathobiology, Ministry of Education, Jilin University, Changchun, Jilin, China

**Keywords:** psychiatric disorders, major depressive disorder, dentofacial deformity, Mendelian randomization, causal relationship

## Abstract

**Background:**

The association between psychiatric disorders and dentofacial deformities has attracted widespread attention. However, their relationship is currently unclear and controversial.

**Methods:**

A two-sample bidirectional MR analysis was performed to study the causal relationship between dentofacial deformity and eight psychiatric disorders, including major depressive disorder, panic disorder, schizophrenia, bipolar disorder, attention deficit hyperactivity disorder, Alzheimer’s disease, autism spectrum disorder, and neuroticism. Inverse variance weighted, weighted median, MR-Egger regression, weighted mode four methods, and further sensitivity analyses were conducted.

**Results:**

The major depressive disorder affected dentofacial deformity, with an OR = 1.387 (95% CI = 1.181-1.629, *P* = 6.77×10^-5^). No other psychiatric disorders were found to be associated with dentofacial deformity. In turn, dentofacial deformity were associated with neuroticism, with an OR = 1.050 (95% CI = 1.008-1.093, *P* = 0.018). And there was no evidence that dentofacial deformity would increase the risk of other psychiatric disorders.

**Conclusions:**

Major depressive disorder might elevate the risk of dentofacial deformities, and dentofacial deformity conditions would increase the risk of the incidence of neuroticism.

## 1 Introduction

The importance of mental health has progressively come to light in the past decades. Depression, as the most prevalent psychiatric disorder, ranks as the third leading cause of the global burden of disease (1). Psychiatric disorders have been identified to affect various conditions, including cardiovascular diseases, bone and joint diseases, and endocrine disorders (2–4). The association between psychiatric disorders and oral diseases has also attract widespread attention (5–7). Dentofacial deformity including malocclusion, is one of the most common oral diseases, affecting mastication, pronunciation, and systemic metabolism in 56% of people worldwide (8–10). Additionally, social responses conditioned by dental appearance also impact the psychological status (11). Patients with severe dental deformities are at a higher risk of suffering from mental illnesses, such as anxiety and depression (12). On the other hand, psychiatric patients exhibit metabolic and behavioral abnormalities that increase the risk of dentofacial deformities. A higher incidence of malocclusions was reported in children with autism and in those who exhibit signs of hyperactivity (13, 14). However, current research on the link between psychiatric disorders and dentofacial deformities is not as abundant as that for other oral disorders, such as periodontitis (15–17). Additionally, the conclusions of existing studies on their relationship remain controversial (18, 19).

This study aims to strengthen and expand understanding of the causal relationship between psychiatric disorders and dentofacial deformity using a bidirectional two-sample Mendelian Randomization (MR) analysis. Eight psychiatric disorders with high prevalence and close relation to oral diseases were selected, including major depressive disorder (MDD), panic disorder (PD), schizophrenia (SCZ), bipolar disorder (BIP), attention deficit hyperactivity disorder (ADHD), Alzheimer’s disease (ALZ), autism spectrum disorder (ASD), and neuroticism. The findings indicate that MDD may increase the risk of dentofacial deformities, and dentofacial deformities could lead to higher incidences of neuroticism. This genetic evidence further reinforces the connection between psychiatric disorders and dentofacial deformities.

## 2 Methods

### 2.1 Experimental design and data sources

A bidirectional study was conducted to clarify the causal relationship between psychiatric disorders and dentofacial deformity. No additional ethical approval or participant consent was required as publicly available data was analyzed in this study. Eight common psychiatric disorders were selected for inclusion, whose genetic associations were obtained from the largest meta-analysis of GWAS conducted by the UK Biobank (UKB) and Psychiatric Genomics Consortium (PGC) (**Table 1**). GWAS statistics for dentofacial deformity (including malocclusion) were obtained from FinnGen consortium R9 release data, including 9866 cases and 259234 controls (28). All study participants were of European ancestry, which helps to avoid bias due to ethnic differences. Additionally, exposure and outcome data were obtained from distinct databases, effectively reducing sample overlap.

**Table 1:** The GWAS consortiums for eight psychiatric traits.

Trait	Data source	nCase	nControl	References
MDD	PGC	246,363	561,190	Genome-wide meta-analysis of depression identifies 102 independent variants and highlights the importance of the prefrontal brain regions (20)
PD	PGC	2248	7992	Genome-wide association study of panic disorder reveals genetic overlap with neuroticism and depression (21)
ADHD	PGC	38,691	186,843	Genome-wide analyses of ADHD identify 27 risk loci, refine the genetic architecture, and implicate several cognitive domains (22)
ASD	PGC	18,382	27,969	Identification of common genetic risk variants for autism spectrum disorder (23)
Neuroticism	UKBB	393411		Mixed-model association for biobank-scale datasets (24)
BIP	PGC	41,917	371,549	Genome-wide association study of more than 40,000 bipolar disorder cases provides new insights into the underlying biology (25)
SCZ	PGC	76,755	243,649	Mapping genomic loci implicates genes and synaptic biology in schizophrenia (26)
ALZ	UKBB	39,106	46,828	New insights into the genetic etiology of Alzheimer’s disease and related dementias (27)

MDD, major depressive disorder; PD, panic disorder; ADHD, attention deficit hyperactivity disorder; ASD, autism spectrum disorder; BIP, bipolar disorder; SCZ, schizophrenia; ALZ, Alzheimer’s disease; UKB, UK Biobank; PGC, Psychiatric Genomics Consortium.

### 2.2 Selection of genetic variables

**Figure 1:**
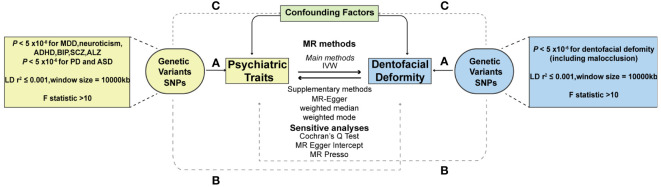
Schematic diagram of two-sample Mendelian randomization. **(A)** The association assumption; **(B)** The exclusion limitation assumption; **(C)** The independence assumption. MR, Mendelian randomization; SNP, single nucleotide polymorphism; IVW, inverse variance weighted; MDD, major depressive disorder; PD, panic disorder; ADHD, attention deficit hyperactivity disorder; ASD, autism spectrum disorder; BIP, bipolar disorder; SCZ, schizophrenia; ALZ, Alzheimer’s disease.

To ensure the validity of the MR analysis, the selection of instrumental variables (IVs) should meet three conditions: (1) the association assumption: the IVs should be strongly correlated with the exposure of interest, (2) the exclusion limitation assumption: the IVs should only affect outcome diseases through exposure, excluding any other pathways, and (3) the independence assumption: the IVs should be independent of any confounding factors (29) (**Figure 1**). For most exposures, the conventional GWAS significance threshold of *P* < 5×10^-8^ was used as the screening condition. However, when this threshold could not provide enough single nucleotide polymorphisms (SNPs), the extended screening condition to *P* < 5×10^-6^ was used for some exposures (PD, ASD, dentofacial deformity). Meanwhile, standard clumping parameters (r^2^ ≤ 0.001; clumping window = 10 000 kb) were used to remove the chain imbalance and filter out independent SNPs (30). To reduce the weak instrumental variable bias, we calculated the F-statistic of the screened independent SNPs [F = R^2^ × (N−2)/(1−R^2^)] and excluded the weakly instrumented variables with F-statistics less than 10 (31).

### 2.3 MR analysis

The analysis of this study was based on the R package “Two Sample MR”. The inverse variance weighted (IVW) method was chosen as the primary analysis method. Weighted median, MR-Egger regression, and weighted mode methods were used as the auxiliary analysis methods. IVW enables the most precise statistics by combining the Wald ratio estimates results for each valid SNP’s causal effect while disregarding invalid IVs and pleiotropy (32). Therefore, as a complement, weighted median, MR-Egger regression, and weighted mode were used to analyze different dimensions of horizontal pleiotropy of IVs. The statistical significance of the *P* value was set at 0.05 (two-sided), and for noncontinuous variables, odds ratios (OR) and 95% confidence intervals (CI), were used to analyze the association between exposure and outcome. The MR-Steiger test was also applied to confirm the directionality that the exposure influenced the results. In addition, Bonferroni correction was applied to the *P* values when multiple factors were included as exposures for analysis.

### 2.4 Sensitivity analysis

To confirm the heterogeneity of MR analysis, Cochran’s Q test was selected, the *P* value of which could demonstrate the existence of heterogeneity. For the studies with heterogeneity (Cochran’s Q test *P* value < 0.05), IVW analysis was a good solution. MR-Egger intercept analysis was used to describe the horizontal pleiotropy of the analysis model. In addition, the MR-PRESSO method could also detect horizontal pleiotropy and simultaneously remove the outliers for correction (33, 34). Also, to verify the stability of the results, a leave-one-out analysis was performed for each SNP.

## 3 Results

### 3.1 Major depressive disorder increases the risk of dentofacial deformity

**Figure 2:**
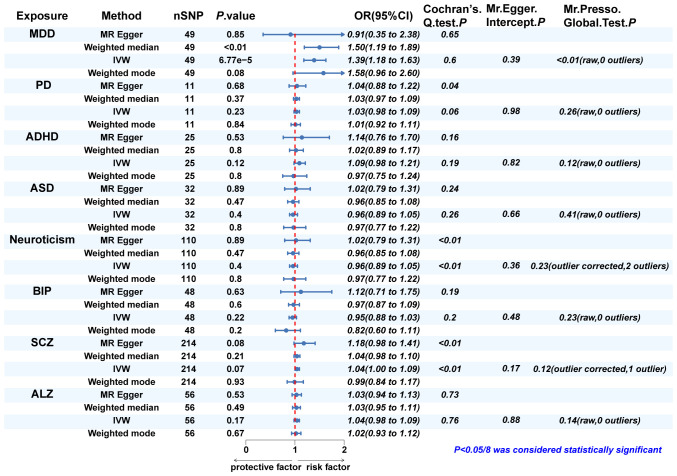
The MR analysis and sensitivity analysis results of psychiatric disorders on dentofacial. deformity. IVW, inverse variance weighted; MDD, major depressive disorder; PD, panic disorder; ADHD, attention deficit hyperactivity disorder; ASD, autism spectrum disorder; BIP, bipolar disorder; SCZ, schizophrenia; ALZ, Alzheimer’s disease.

**Figure 3:**
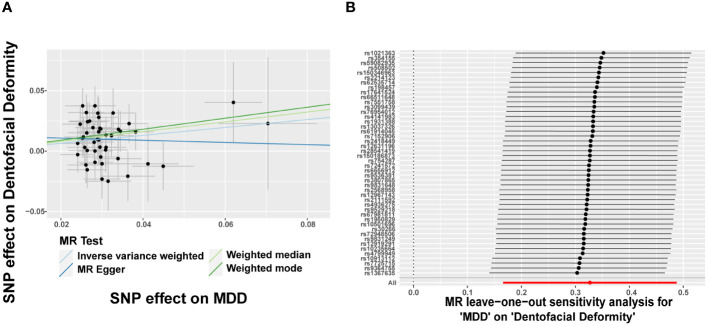
Major depressive disorder increases the risk of dentofacial deformity. **(A)** Scatter plots for the effect of MDD on risk of dentofacial deformity, **(B)** Forest plot of the results of the leave-one-out sensitivity analysis on the effect of MDD on dentofacial deformity. SNP, single nucleotide polymorphism; MDD, major depressive disorder.

Since eight psychiatric disorders were selected as the exposure factors in the study, Bonferroni correction was additionally performed to improve accuracy. A causal relationship was considered statistically significant when the corrected *P* value was less than 0.0065 (0.05/8). The effects of eight psychiatric disorders on dentofacial deformity were investigated by four methods mentioned above (**Figure 2**). In the main IVW study, MDD increased the risk of developing dentofacial deformity, with an OR = 1.387 (95% CI = 1.181-1.629, *P* = 6.77×10^-5^) (**Figure 3A**). The correct causal direction (*P* = 1.122352e^-50^) was ensured through the MR-Steiger test. Both the heterogeneity and horizontal pleiotropy of MDD effects on dentofacial deformity were rejected in Cochran’s Q test and MR-Egger intercept analysis. Moreover, the MR-PRESSO test did not find the outliers (**Figure 2**). In addition, the leave-one-out analysis excluded the extreme influence of one single SNP (**Figure 3B**). Beyond, statistically, several other psychiatric disorders did not influence the incidence of dentofacial deformity statistically (**Supplementary Figure S1**).

### 3.2 Dentofacial deformity increases the risk of neuroticism

**Figure 4:**
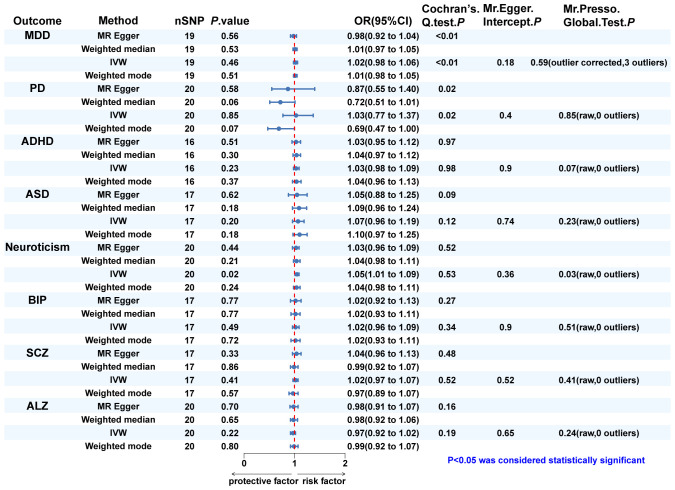
The MR analysis and sensitivity analysis results of dentofacial deformity on psychiatric. disorders. IVW, inverse variance weighted; MDD, major depressive disorder; PD, panic disorder; ADHD, attention deficit hyperactivity disorder; ASD, autism spectrum disorder; BIP, bipolar disorder; SCZ, schizophrenia; ALZ, Alzheimer’s disease.

**Figure 5:**
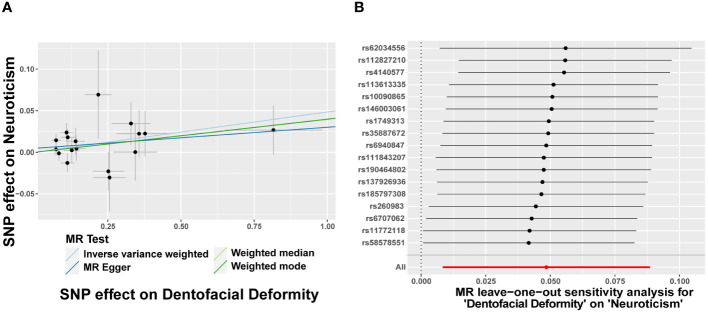
Dentofacial deformity increases the risk of neuroticism. **(A)** Scatter plots for the effect of dentofacial deformity on risk of neuroticism, **(B)** Forest plot of the results of the leave-one-out sensitivity analysis on the effect of dentofacial deformity on neuroticism. SNP, single nucleotide polymorphism.

In turn, dentofacial deformity was used as the exposure to study the effects on psychiatric disorders. Respectively, there was an association between the presence of dentofacial deformity and the occurrence of neuroticism (**Figure 4**), with an OR = 1.050 (95% CI = 1.008-1.093, *P* = 0.018) in the IVW analysis (**Figure 5A**). Similarly, the stability of the results was verified by sensitivity analysis. The heterogeneity, the horizontal pleiotropy, and the extreme effect of one single SNP were all rejected (**Figures 4**, **5B**). Additionally, the MR-Steiger test ruled out the possibility of reverse causation (*P* = 7.480458e^-39^). Other than this, no statistical association was found between dentofacial deformity and other psychiatric disorders (**Supplementary Figure S1**).

### 3.3 Sensitivity analysis

In Cochran’s Q test, heterogeneity was rejected for most of the studies, except for the effects of SCZ and neuroticism on dentofacial deformity and the effects of dentofacial deformity on MDD (**Figures 2**, **4**). The random effects model for IVW analysis was a suitable choice for the studies with mentioned heterogeneity. As for horizontal pleiotropy, it was rejected in all the studies in MR-Egger intercept test. For the studies in which outliers were found, the analysis was repeated after excluding the outliers in the MR-PRESSO test (**Figures 2**, **4**). Leave-one-out analysis confirmed that no extreme SNP affected the results in any study (**Supplementary Figure S2**).

## 4 Discussion

The MR analysis can be used to study the causal relationship between two complex diseases by selecting SNPs, which are highly correlated with the exposure diseases, as IVs to observe their effects on the outcome diseases. It may be seen as a kind of randomized clinical trial (RCT) since the SNPs are randomly assigned between parents and offspring, effectively lowering the influence of confounding variables and avoiding reverse causation bias compared to other observational line research methods (35). Here, a bidirectional two-sample MR analysis was carried out to study the casual relationship between psychiatric disorders and dentofacial deformities. Specifically, MDD elevated the risk of dentofacial deformities, while dentofacial deformity conditions also increased the incidence of neuroticism.

A growing body of evidence indicates that MDD may be the risk factors for dentofacial deformities by affecting systemic bone metabolism. Patients with depression typically exhibit dysfunction of the hypothalamic-pituitary-adrenal (HPA) axis, elevated concentrations of adrenocorticotropic hormone (ACTH) and cortisol, increased production of inflammatory factors, and upregulation of osteoclast activity, ultimately resulting in aberrant bone metabolism (36). Consequently, impaired skeletal development, including anomalies in dentofacial growth, is commonly noted in adolescents with depression (37, 38), reflecting disturbances in bone metabolism during critical growth periods. Additionally, depressive disorder impacts embryonic dentofacial development. Evidence suggests depression during pregnancy has a detrimental effect on fetal growth and development (39), and increase the risk of dentofacial deformities in offspring (40).

In addition to MDD itself, ongoing antidepressant therapy is also a risk factor for dentofacial deformities. The most widely used antidepressants for depression, selective serotonin reuptake inhibitors (SSRIs), have been shown to impair bone metabolism (36), since elevated serotonin levels caused by SSRIs may prevent osteoblastic cells from proliferating, differentiating, and mineralizing (41). Variations in serotonin levels are associated with anatomical and physiological abnormalities *in utero*, resulting in craniofacial development anomalies such as cleft palate, craniosynostosis, and dental deformities (42). Additionally, low postnatal weight, short length, small head circumference, and developmental growth retardation were observed in SSRI-exposed intrauterine fetuses or breastfed babies as a result of maternal drug use (39, 43). Hence, MDD and the intake of antidepressants can similarly obstruct dentofacial development especially in adolescence and embryonic stage, thus elevating the risk of dentofacial deformities.

Previous studies have also indicated that psychiatric disorders other than MDD may have effect on the dentofacial deformities, but this remains controversial. Like children with ASD or ADHD may exhibit significantly increased abnormal behavioral patterns, such as finger-sucking and lip-biting, which can contribute to the occurrence and progression of dentofacial deformities (13, 14). Nevertheless, a recently published systematic review suggested insufficient evidence to associate the prevalence of dentofacial deformities with ASD or ADHD (19). Additionally, olanzapine, a commonly used drug for SCZ, has been shown to inhibit osteoblast function and obstruct skeletal development by down-regulating the Wnt/ß-catenin pathway (44). However, other studies have shown that olanzapine usage does not result in bone metabolism abnormalities (45). Similarly, there was no effect of other psychiatric disorders except MDD on dentofacial deformities found in this MR study.

According to popular belief, patients with dentofacial deformities are thought to be more vulnerable to psychological distress due to a lack of confidence in facial features, which may result in depression or anxiety (46). Though a few studies provided support for this viewpoint (12), the majority of research found no evidence to support the perspective that dentofacial deformities could increase the risk of psychiatric disorders (18, 47). Rather than depression or anxiety, this MR study indicated that dentofacial deformity was a risk factor for neuroticism. Considering that patients who require orthognathic surgical intervention due to severe dentofacial deformities are comparatively more susceptible to psychological distress (47), that combining dentofacial deformities of varying severity for analysis may get unclear results. Further analysis of dentofacial deformity subgroups that vary in severity may provide a comprehensive understanding of its association with psychiatric disorders.

Among the eight psychiatric disorders involved in this study, ALZ is more widely recognized as being associated with oral diseases, especially periodontitis (48). By elevating the degree of systemic inflammation, periodontitis was demonstrated to contribute to the development of Alzheimer’s cognitive impairment (49, 50). Since malocclusion is considered a susceptibility factor for periodontitis by challenging oral cleaning (51), the potential impact of dentofacial deformities on ALZ was evaluated. At first, a positive relationship was observed when a small sample GWAS database from PGC (52) was selected for analysis (data not shown). However, no effect of dentofacial deformity on ALZ was shown when another more extensive database was applied. Although a clear association between dentofacial deformities and ALZ cannot yet be established, further investigations may provide new insight into the possibility that these two conditions are linked through periodontitis.

Actually, in addition to the above-discussed bidirectional causative relationship, psychiatric disorders, and dentofacial skeletal development may have a considerably stronger correlation than is currently recognized. More recently, the novel role of osteogenic proteins associated with dentofacial development, such as bone morphogenetic protein (BMP) (53) and neural EGFL-like 1 (Nell-1) (54), in the central nervous system has been revealed. BMP increased in the hippocampus of mice under psychological stress (55), and BMP signaling inhibition showed antidepressant properties (56). Nell-1-haploinsufficient (*Nell-1^+/6R^*) mice display core abnormalities similar to autism spectrum disorder (57). A closer connection between psychiatric disorders and dentofacial deformities comes to light due to the identification of these multifunctional proteins affecting both bone development and nerve function, which also broadens the scope of future studies on their relationship.

This study reinforces the connection between psychiatric disorders and dentofacial deformities, advances our understanding of the etiology of dentofacial deformities, and highlights the necessity of continuous psychological condition monitoring during orthodontic treatment. However, the study still has some limitations, as reflected in the following. First, the limited sample size of dentofacial deformity GWAS data restricted the selection of genetic variables. Second, only the population of European origin was analyzed because of sample source limitations. Third, there was no subgroup analysis of the different types or severity of dentofacial deformities. Together with our findings, these currently intractable limitations offer additional opportunities and targets for further in-depth study, especially subgroup analyses on sizable samples.

## 5 Conclusion

MDD works as a risk factor for dentofacial deformity. Dentofacial deformity contributes to the chance of neuroticism.

## Data availability statement

The original contributions presented in the study are included in the article/**Supplementary Material**. Further inquiries can be directed to the corresponding authors.

## Author contributions

JN: Conceptualization, Data curation, Formal analysis, Writing – original draft, Writing – review & editing. YZ: Conceptualization, Formal analysis, Methodology, Writing – review & editing. JM: Conceptualization, Data curation, Formal analysis, Methodology, Writing – review & editing. QX: Conceptualization, Data curation, Software, Writing – original draft. MH: Funding acquisition, Supervision, Writing – review & editing. HQ: Funding acquisition, Supervision, Validation, Writing – review & editing.
