# Supplementary material for: Major depressive disorder elevates the risk of dentofacial deformity: a bidirectional two-sample Mendelian randomization study
Source: Front Psychiatry. 2024 Jul 30;15:1442679. doi: 10.3389/fpsyt.2024.1442679 (PMC11319251; doi:10.3389/fpsyt.2024.1442679)
Supplement: Supplementary file 1 [file DataSheet_1.docx]

Supplementary Material

## Supplementary Figures


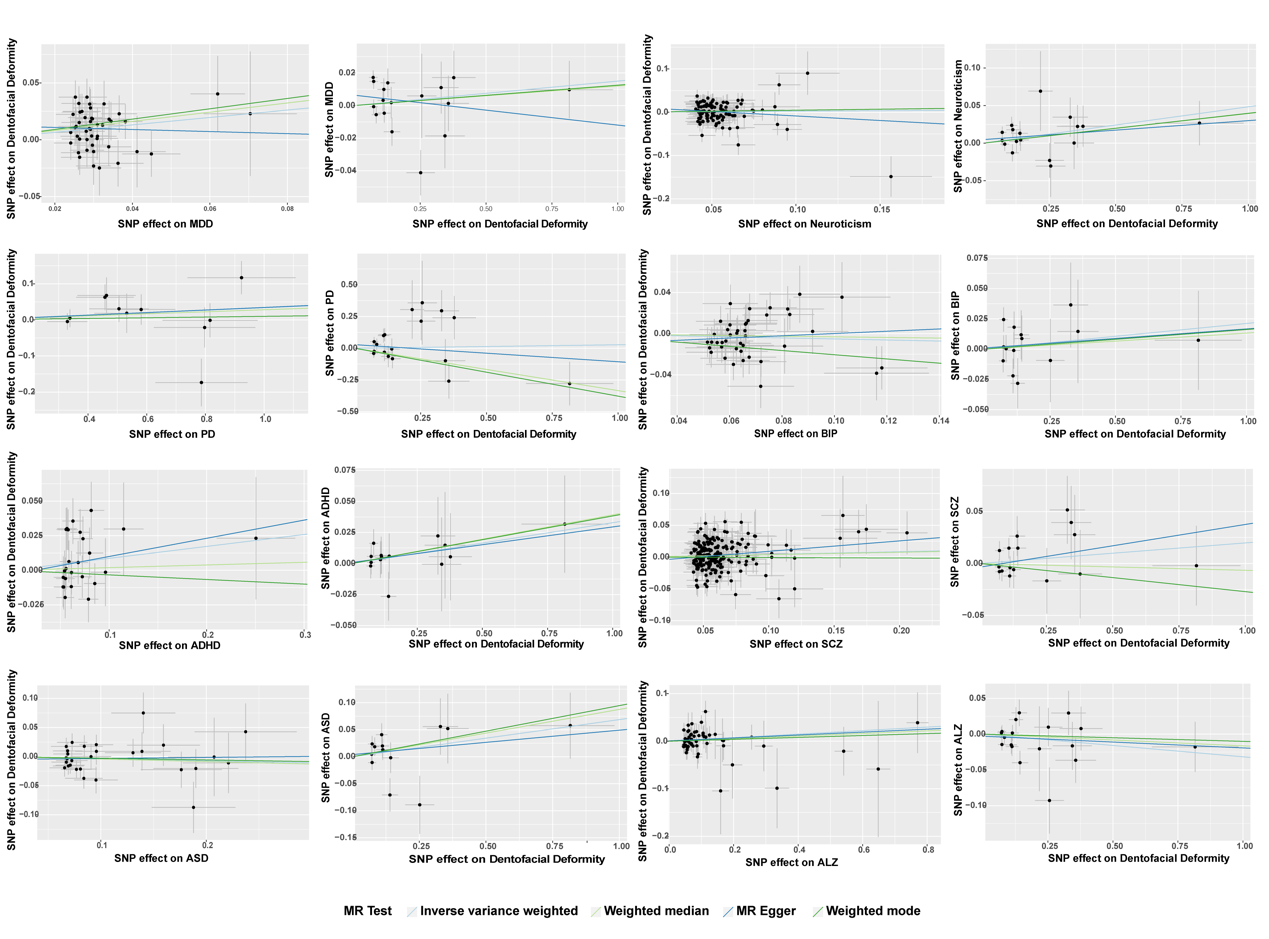


**Supplementary Figure 1. Scatter plots of the MR results.**


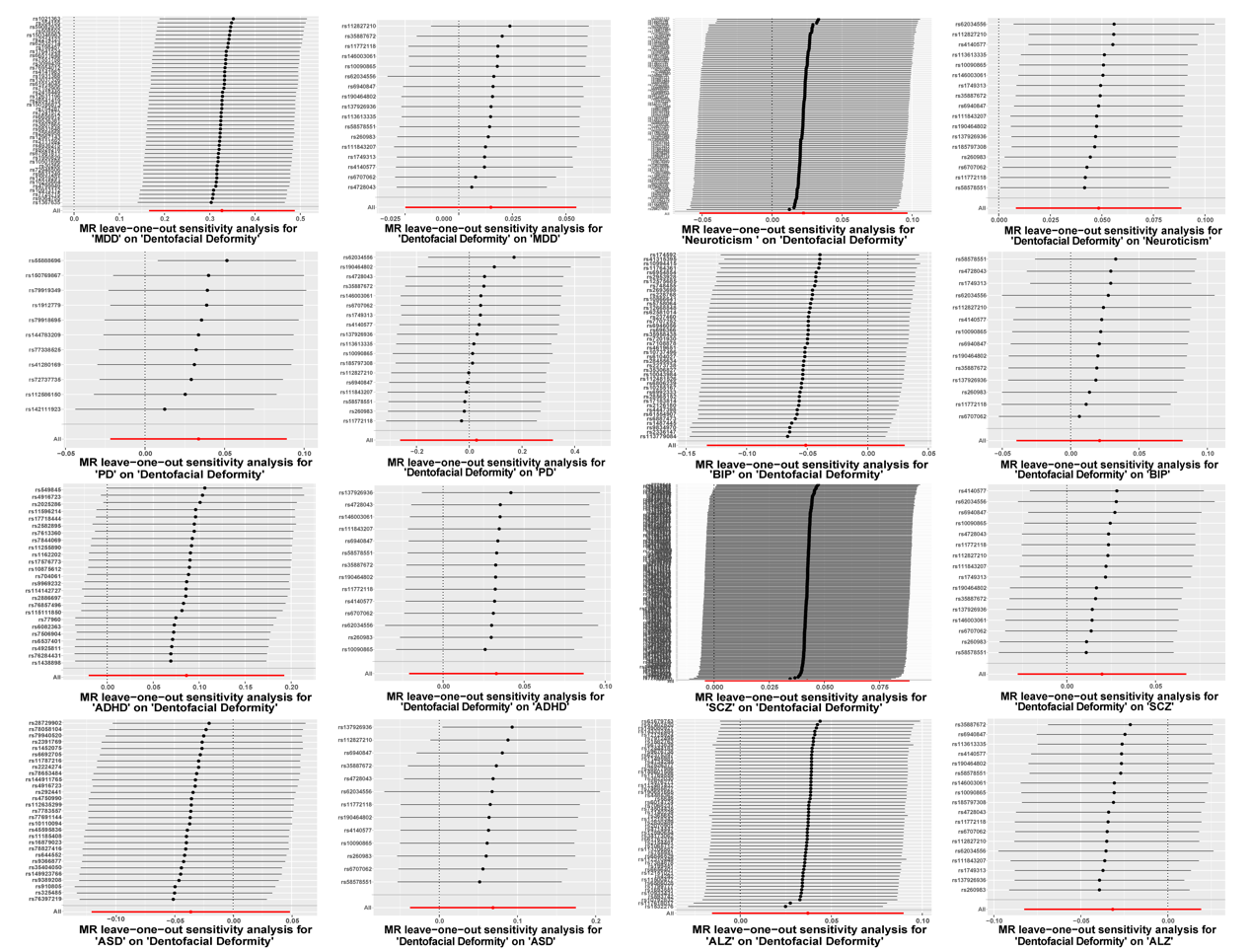


**Supplementary Figure 2. Forest plot of leave-one-out analysis.**
